# Circadian rhythm influences genome-wide transcriptional responses to ^131^I in a tissue-specific manner in mice

**DOI:** 10.1186/s13550-015-0150-y

**Published:** 2015-12-15

**Authors:** Britta Langen, Nils Rudqvist, Toshima Z. Parris, Khalil Helou, Eva Forssell-Aronsson

**Affiliations:** Department of Radiation Physics, Institute of Clinical Sciences, Sahlgrenska Cancer Center, Sahlgrenska Academy, University of Gothenburg, SE-413 45 Gothenburg, Sweden; Department of Applied Physics, Chalmers University of Technology, Gothenburg, Sweden; Department of Oncology, Institute of Clinical Sciences, Sahlgrenska Cancer Center, Sahlgrenska Academy, University of Gothenburg, Gothenburg, Sweden; Department of Medical Physics and Biomedical Engineering, Sahlgrenska University Hospital, Gothenburg, Sweden

**Keywords:** Iodine-131, Normal tissue response, Circadian rhythm, Microarray, Radiogenomics

## Abstract

**Background:**

Circadian variation of gene expression is often neglected when ionizing radiation-induced effects are studied, whether in animal models or in cell culture. This study characterized diurnal variation of genome-wide transcriptional regulation and responses of potential biomarkers and signature genes in normal mouse tissues at 24 h after i.v. administration of ^131^I.

**Methods:**

Female BALB/c nude mice were i.v. injected with 90 kBq ^131^I at 9:00 a.m., 12:00 p.m., or 3:00 p.m. and killed after 24 h (*n* = 4/group). Paired control groups were mock-treated (n = 3–4/group). The kidneys, liver, lungs, spleen, and thyroid were excised, snap-frozen, and stored at −80 °C until extraction of total RNA. RNA microarray technology was used for genome-wide expression analysis. Enriched biological processes were categorized after cellular function. Signature genes for ionizing radiation and thyroid hormone-induced responses were taken from the literature. Absorbed dose was estimated using the Medical Internal Radiation Dose (MIRD) formalism.

**Results:**

The thyroid received an absorbed dose of 5.9 Gy and non-thyroid tissues received 0.75–2.2 mGy over 24 h. A distinct peak in the total number of significantly regulated transcripts was observed at 9:00 a.m. in the thyroid, but 3 h later in the kidney cortex, kidney medulla, and liver. Transcriptional regulation in the lungs and spleen was marginal. Associated cellular functions generally varied in quality and response strength between morning, noon, and afternoon. In the thyroid, 25 genes were significantly regulated at all investigated times of day, and 24 thereof showed a distinct pattern of pronounced down-regulation at 9:00 a.m. and comparatively weak up-regulation at later times. Eleven of these genes belonged to the species-specific kallikrein subfamily *Klk1b*. Responses in signature genes for thyroid hormone-induced responses were more frequent than for ionizing radiation, and trends persisted irrespective of time of day.

**Conclusion:**

Diurnal variation of genome-wide transcriptional responses to 90 kBq ^131^I was demonstrated for the thyroid, kidney cortex and medulla, and liver, whereas variation was only marginal in the lungs and spleen. Overall, significant detection of potential biomarkers and signature genes was validated at each time of day, although direction of regulation and fold-change differed between morning, noon, and afternoon. These findings suggest that circadian rhythm should be considered in radiation research and that biological and analytical endpoints should be validated for circadian robustness.

**Electronic supplementary material:**

The online version of this article (doi:10.1186/s13550-015-0150-y) contains supplementary material, which is available to authorized users.

## Background

Circadian rhythmicity is an intrinsic variable when studying responses in living organisms. In most species—including not only animals but also plants and microbes—many cellular, physiological, and behavioral processes are regulated with 24-h periodicity by endogenous pacemakers or exogenous zeitgeber signals [1]. Circadian rhythm is governed mainly by the suprachiasmatic nucleus (SCN) and molecular clock genes, but the molecular mechanisms of circadian rhythm in peripheral tissues are not yet fully elucidated [2–5]. When studying effects of ionizing radiation (IR) exposure in normal tissue, circadian variation constitutes an unknown variable if it is not controlled in the experimental design. It has been shown that circadian rhythm controls oscillation of gene expression on both the genetic and proteomic level and thus regulates tissue function in relation to the time of day [6, 7]. Accordingly, research to identify molecular biomarkers or to characterize genome-wide normal tissue responses after IR exposure needs to consider circadian rhythm as an experimental variable.

Various studies have been performed to identify IR-associated signatures, most of them using in vitro model systems [8, 9]. To the best of our knowledge, however, robustness of molecular biomarkers for ionizing radiation exposure in vivo in relation to time of day has not been investigated yet. Our research group has performed microarray analysis of various normal mouse tissues to characterize transcriptional regulation and to identify potential biomarkers in response to IR exposure, specifically from i.v.-administered radionuclides used in cancer therapy [10–15]. In this study, we analyzed genome-wide transcriptional regulation in the mouse thyroid, kidney cortex and medulla, liver, lungs, and spleen with regard to diurnal variation from morning to afternoon. This period was chosen since it represents day times commonly used in laboratory schedules.

Our group has investigated the impact of systemic effects on transcriptional responses in vivo when a regulatory organ receives a much higher absorbed dose compared with the other tissues in the body ([11], Langen B, Rudqvist N, Helou K, Forssell-Aronsson E: Microarray studies on ^211^At administration in BALB/c nude mice indicate systemic effects on transcriptional regulation in non-thyroid tissues, submitted). ^131^I and ^211^At are taken up by the thyroid to a greater extent than in other tissues [16, 17], which can have an impact on normal thyroid function. It has been shown that ^131^I and ^211^At exposure has effects on iodide transport and on mRNA expression of the sodium-iodide symporter (NIS) in thyroid follicular cells in vitro [18]. In the in vivo setting, 60–1636 transcripts were found to be differentially expressed in response to ^131^I or ^211^At exposure [10, 13]. Furthermore, upon i.v. administration in vivo, IR-induced effects in the thyroid are hypothesized to influence transcriptional responses in the kidneys, liver, lungs, and spleen to varying extent ([11], Langen B, Rudqvist N, Helou K, Forssell-Aronsson E: Microarray studies on ^211^At administration in BALB/c nude mice indicate systemic effects on transcriptional regulation in non-thyroid tissues, submitted).

The aim of this study was to evaluate the impact that time of day may have on ^131^I-induced effects in the mouse thyroid, kidney cortex and medulla, liver, lungs, and spleen. In order to avoid limitation to a presupposed set of genes or IR-induced signaling pathways, we used RNA microarray technology to analyze induced transcriptional regulation on a genome-wide scale. We also analyzed differential regulation of signature genes for IR-associated and thyroid hormone (TH)-induced responses respective to time of day in order to evaluate the potential systemic impact from the thyroid on gene regulation in non-thyroid tissues.

## Methods

### Radionuclide administration and radioactivity measurements

^131^I was obtained from GE Healthcare (Braunschweig, Germany) in the form of NaI. Syringes were weighed before and after injection. The gamma counter Wallac 1480 Wizard® 3" (Wallac Oy; Turku, Finland) was used to measure ^131^I activity in syringes before and after administration. The mean injected activity was corrected for the mean residual activity after administration.

### Estimation of absorbed dose

The Medical Internal Radiation Dose (MIRD) formalism

$$ {\overline{D}}_{\mathrm{organ}}=\frac{{\tilde{A}}_{\mathrm{organ}} \times {\displaystyle {\sum}_i}{n}_i\ {E}_i{\varPhi}_i}{m_{\mathrm{organ}}}, $$

was used to calculate mean absorbed dose for each organ $\left({\overline{D}}_{\mathrm{organ}}\right)$ assuming a homogeneous activity distribution with yield *n*_*i*_ of radiation *i* with energy *E*_*i*_ and absorbed fraction *Φ*_*i*_ in the target organ with mass *m*_organ_ [19]. *Ã*_organ_ designates the time-integrated ^131^I activity in the organ estimated with the trapezoidal rule from 0 to 24 h based on previously reported biodistribution data [20]. For the kidneys, spleen, and thyroid, electron emission was only considered from ^131^I located in respective tissues, and *Φ*_*i*_ was set to 0.919, 0.854, and 0.742, respectively [21]. For the liver, electrons emitted from ^131^I located in the lungs were also considered, and *Φ*_*i* liver←liver_ was set to 0.954, and *Φ*_*i* liver←lungs_ was set to 0.011 [21]. Likewise, the liver was considered as a source organ for lung absorbed dose calculation, and *Φ*_*i* lungs←lungs_ was set to 0.85, and *Φ*_*i* lungs←liver_ was set to 0.008 [21].

### Animal experiments

Female BALB/c nude mice (CAnN.Cg-Foxn1nu/Crl; Charles River Laboratories International, Inc.; Salzfeld, Germany) were chosen for these experiments, because they are frequently used for establishing human tumor xenografts as an in vivo model for radionuclide therapy. Accordingly, the effect of circadian rhythm was investigated in the model system commonly used in pre-clinical studies. Nine-month-old female BALB/c nude mice were injected into the tail vein with 90 kBq ^131^I in 0.15 ml physiological saline. The mean mouse weight was approximately 21.8 ± 2.2 g. Administrations were performed at 9:00 a.m., 12:00 p.m., and 3:00 p.m. (*n* = 4/group), hereafter referred to as 9:00, 12:00, and 15:00, with an individual control group for each time of day. Control groups were mock-treated with physiological saline (*n* = 3–4/group). Physiological saline as control solution is considered adequate in this set-up, since the injected amount of iodine atoms is roughly 10^6^ times lower than the daily intake of stable iodine-127 (^127^I) from standard chow, and accordingly, the effect from iodine administration itself is considered negligible. In total, 22 mice were used in this study, i.e, 12 mice injected with ^131^I and 10 mice used as controls. These times of day were chosen since they represent working hours commonly used in animal experiments. Animals were kept in individually ventilated cages in groups between 6 to 10 animals per cage, and health status was monitored routinely. Animals were kept at standard dark/night cycle with water and food available ad libitum. At the day of injection, animals were kept in the dark from 5:00 p.m. to 8:30 a.m. the following day and experimental groups were kept in individual cages in a radiation safety-monitored animal laboratory. At 24 h after respective administrations, animals were anesthetized with sodium pentobarbital and killed via cardiac puncture. The kidneys, liver, lungs, spleen, and thyroid were excised, flash-frozen, and stored at −80 °C until analysis. All animal procedures were approved by the Ethical Committee on Animal Experiments in Gothenburg, Sweden (registration number 188/11).

### Gene expression analysis

Individual tissue samples were prepared from excised organs, and total RNA was extracted according to established procedures [11]. Concentration and RIN value of RNA samples were determined as described previously [11]. All samples had a RIN value of at least 6.0 and were analyzed on Illumina MouseRef-8 Whole-Genome Expression BeadChips (Illumina; San Diego, CA, USA) at the Swegene Center for Integrative Biology Genomics DNA Microarray Resource Center (SCIBLU; Lund, Sweden). The Illumina and BioArray Software Environment (BASE; SCIBLU) was used for image acquisition, raw signal quantification, data preprocessing, and quantile normalization as described previously [11]. Subsequent data processing was performed with Nexus Expression 3.0 (BioDiscovery; El Segundo, CA, USA) as described elsewhere [22]. The Benjamini-Hochberg method was used to control false discovery rate (FDR) in comparison analysis between microarray data sets with an adjusted *p* value cutoff of 0.01 [23]. Significantly regulated transcripts were identified with a fold-change threshold of at least 1.5, hereafter referred to as (differentially) *regulated* transcripts. The gene expression data in this study have been deposited in NCBI’s Gene Expression Omnibus (GEO accession GSE66303).

Literature-based signature genes were used to discern between IR-associated (56 genes) and TH-induced (61 genes) transcriptional responses as described elsewhere [Langen B, Rudqvist N, Helou K, Forssell-Aronsson E: Microarray studies on ^211^At administration in BALB/c nude mice indicate systemic effects on transcriptional regulation in non-thyroid tissues, submitted]. Regulation of circadian clock core genes was analyzed to evaluate the effect of 90 kBq ^131^I on the molecular components of circadian rhythm regulation (Additional file 1: Table S1) [24]. Transcript-associated Gene Ontology (GO) terms were enriched for biological processes, and statistical significance was accepted with an adjusted *p* value of less than 0.05 [25]. In order to obtain a comprehensive overview on associated cellular effects, biological processes were categorized into 8 main categories encompassing over 30 subcategories according to the Gene Ontology database (http://www.geneontology.org), as described previously [11]. The strength of response was expressed as the percentage of scored vs. filtered transcripts of all biological processes categorized in a given subcategory (and summarized for respective main category) and visualized as a heat map.

## Results

### Tissue-specific absorbed dose and total transcriptional response

**Fig. 1:**
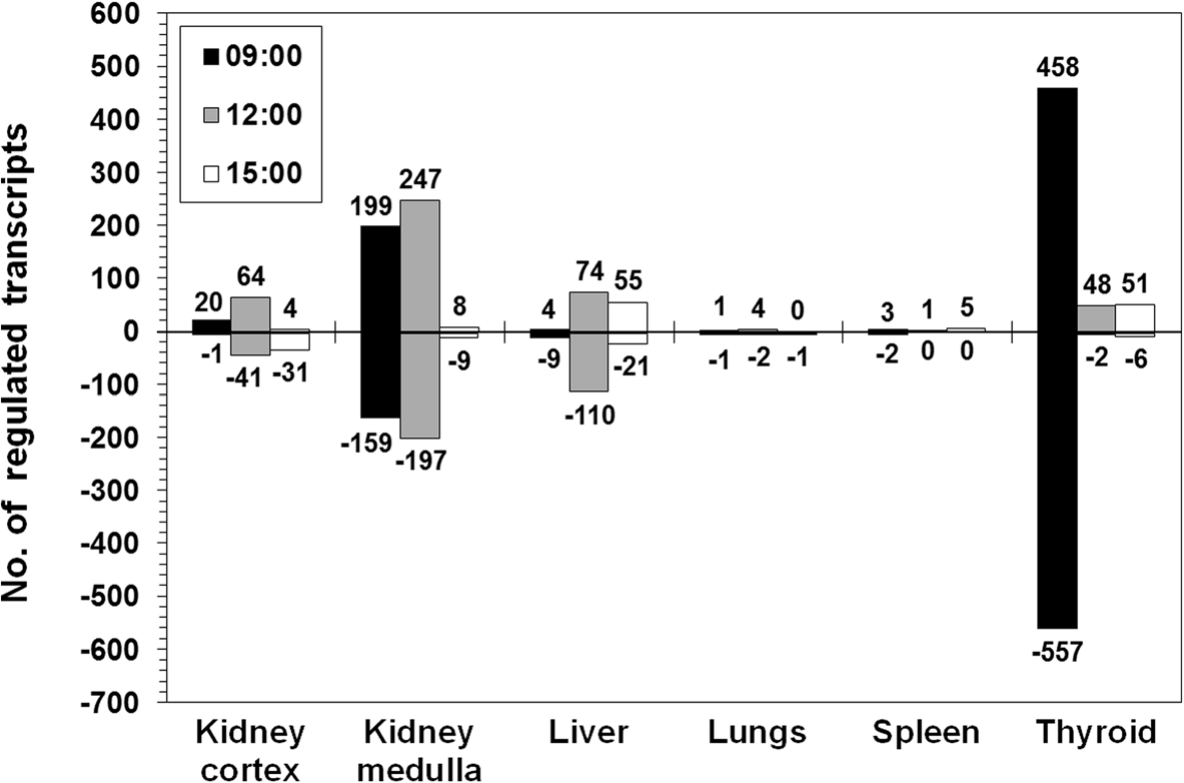
Number of significantly regulated transcripts. The number (*no.*) of significantly up- and down-regulated transcripts (probes) (*positive and negative numbers*, respectively) is shown for each tissue after 24 h following i.v. administration of 90 kBq ^131^I at 9:00 (*black*), 12:00 (*gray*), and 15:00 (*white*)

The absorbed dose from 90 kBq ^131^I was much higher in thyroid tissue than in the investigated non-thyroid tissues. The thyroid received 5.9 Gy over 24 h, while these non-thyroid tissues received between 0.75 and 2.2 mGy (Table 1). Transcript up- and down-regulation differed strongly between the tissues and showed sensitivity to the time of day (Fig. 1). The strongest response was seen in the thyroid at 9:00 with 1015 regulated transcripts. At 12:00 and 15:00, however, transcript regulation was on a much lower level with only 50 and 57 regulated transcripts, respectively. In the non-thyroid tissues, the overall response was strongest in the kidney medulla followed by liver and kidney cortex. In these tissues, a pronounced maximum was observed at 12:00 with 444 (kidney medulla), 184 (liver), and 105 (kidney cortex) regulated transcripts. In contrast, regulation ranged between 1 and 6 transcripts in the lungs and spleen over the course of the investigated times of day.

**Table 1:** Tissue-specific mean absorbed dose

Mean absorbed dose (mGy)				
	Kidney cortex		2.1	
	Kidney medulla		2.1	
	Liver		1.4	
	Lungs		2.2	
	Spleen		0.75	
	Thyroid		5 900	

Mean absorbed dose (mGy) to mouse tissues over 24 h following i.v. administration of 90 kBq ^131^I

### Variation in total transcriptional response through the day

**Fig. 2:**
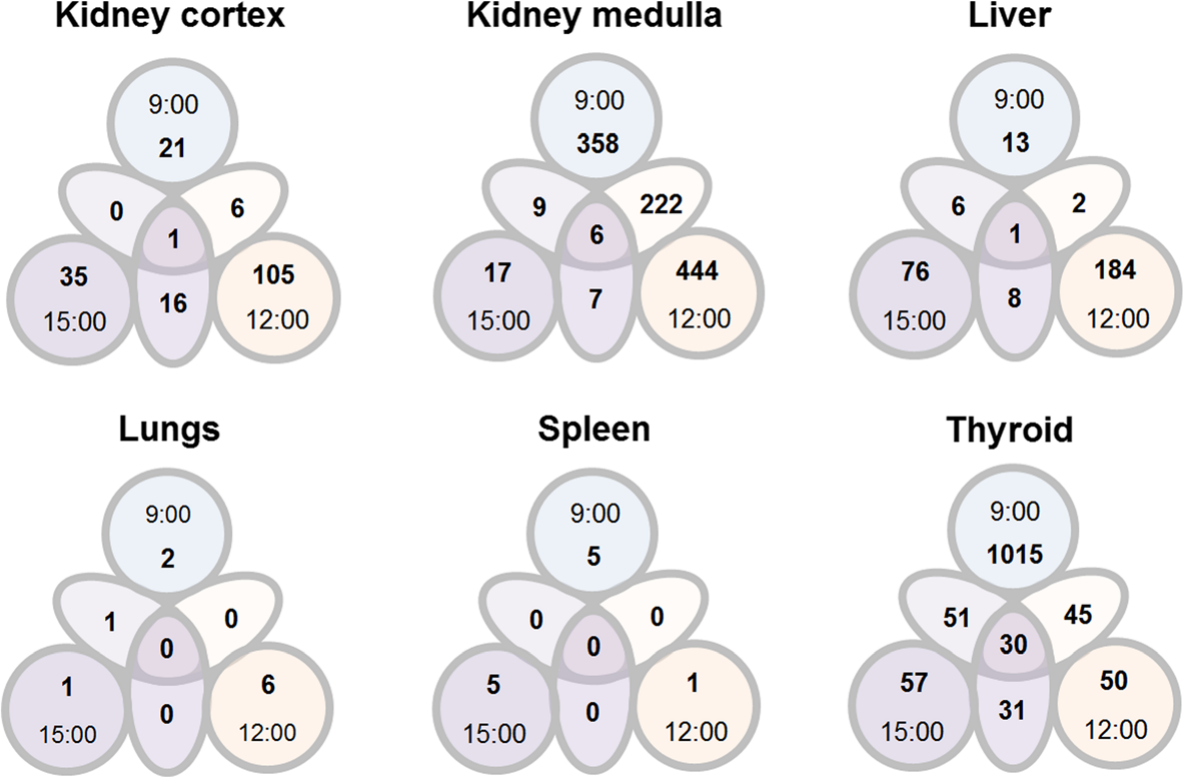
Shared transcript regulation between different day times of administration. The total number (*no.*) of significantly up- and down-regulated transcripts after 24 h following i.v. administration of 90 kBq ^131^I at 9:00, 12:00, and 15:00 is shown. Statistical comparison between exposure group(s) and control group(s) was made individually for each time of day (*large circles*), for pair-wise comparison between time points (*oval shape* between *large circles*), and over all time points (*center circle*). Large circles indicate the number of differentially regulated transcripts in a treated group compared with the control group at the respective time of day. *Oval shapes* between *circles* indicate the number of transcripts that were shared between two time points, i.e., transcripts that were differentially regulated in both treated groups compared with the two respective control groups. The *center circle* indicates the number of transcripts that were differentially regulated and shared among all time points, i.e., comparisons were performed between all three treated groups with their respective control groups. Please note that the diagram is not a classical Venn diagram, since the statistical population differs between each comparison analysis

Thyroid showed the highest relative ratio of differentially regulated transcripts that were shared between two or all three times of day (Fig. 2). The vast majority of the transcripts responding at 12:00 or 15:00 were also regulated at 9:00, i.e., 45 of 50 and 51 of 57 transcripts, respectively; moreover, 30 of these transcripts were regulated at all times of day. In comparison, few transcripts were regulated at all three times of day in the non-thyroid tissues, i.e., six transcripts in the kidney medulla, one transcript in the kidney cortex and the liver, and none in the lungs and spleen—the latter basically due to the generally low response in both tissues (Figs. 1 and 2). Similarly, few transcripts were shared between two times of day in the non-thyroid tissues; a notable exception was seen in the kidney medulla, where over 60 % of regulated transcripts at 9:00 were also regulated at 12:00, which also constituted half of the regulation observed at 12:00.

**Fig. 3:**
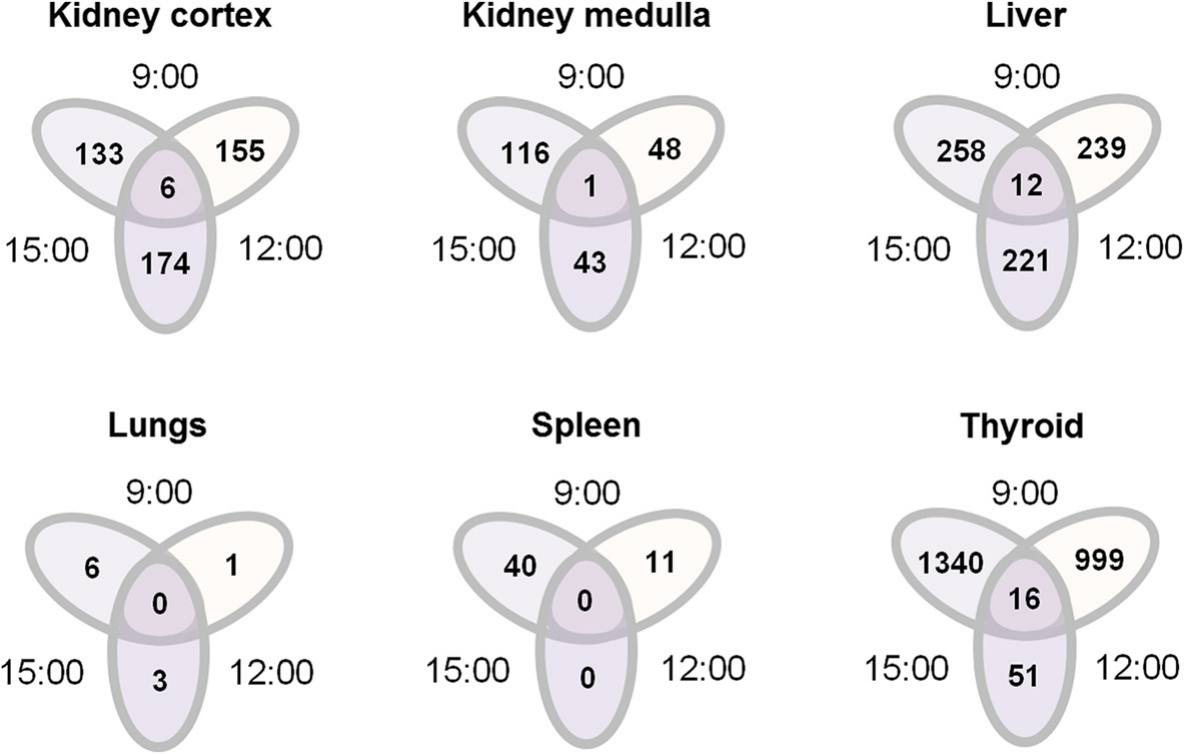
Differential transcript regulation through the day. The total number (*no.*) of differentially regulated transcripts (both significant up- and down-regulation of detected probes) is shown for control groups, i.e., after 24 h following i.v. administration of physiological saline at 9:00, 12:00, and 15:00. Statistical comparisons were made individually for pair-wise comparison between time points and over all three time points. *Ovals* indicate the number of differentially regulated transcripts between two time points; *circles* indicate the number of differentially regulated transcripts shared between all time points. Please note that the diagram is not a classical Venn diagram; for further description, please refer to Fig. 2

The impact that time of day may have as an individual factor on transcriptional regulation was evaluated by a separate statistical comparison analysis between control groups only, i.e., pair-wise comparisons between two times of day and over all three ones (Fig. 3). The extent of diurnal variation in the absence of ^131^I administration also showed tissue-specificity. Moreover, the number of differentially regulated transcripts between two times of day was distinctly higher among control groups than was observed for analysis of ^131^I-treated groups, with the exception of kidney medulla (between 9:00 and 12:00) and spleen (between 12:00 and 15:00) (*cf.* Figs. 2 and 3). The largest number of differentially regulated transcripts was seen in the thyroid between 9:00 and 15:00 with 1340 transcripts followed by 999 transcripts between 9:00 and 12:00 (Fig. 3). In contrast, only 51 transcripts were differentially regulated between 12:00 and 15:00. The second largest difference was seen in the liver with 221–258 differentially regulated transcripts; meaning, in contrast to the thyroid, the extent of diurnal variation did not vary much through the day. Similarly, the kidney cortex did not show a pronounced peak in differential regulation through the day, but the overall response (133–174 transcripts) lay on a somewhat lower level compared with the liver. In the kidney medulla, a maximum of differential regulation was seen between 9:00 and 15:00 with 116 transcripts, while only 43–48 transcripts were differentially regulated between the other times of day. Differential regulation was on a very low level in the lungs for both comparison analyses, i.e., in response to ^131^I administration and in response to diurnal variation alone (*cf.* Figs. 2 and 3). The spleen, however, showed a notable difference between both analyses; no differentially regulated transcripts were shared between time points after ^131^I administration, yet 40 and 11 transcripts were differentially regulated among control groups between 9:00 and 15:00 and between 9:00 and 12:00, respectively (Fig. 3). Similar to ^131^I administration, the number of transcripts that were differentially regulated and shared between all three times of day was comparatively low among control groups in all investigated tissues.

### Potential biomarker genes regulated independent of time of day

**Fig. 4:**
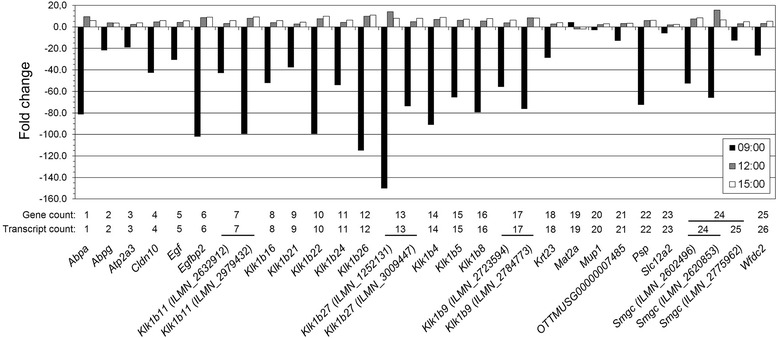
Transcripts regulated over time of day in thyroid. The fold-change of transcripts significantly regulated at all investigated times of day (9:00, *black*; 12:00, *gray*; 15:00, *white*) is shown for the thyroid. Up- and down-regulation is indicated by positive and negative values, respectively. Probe IDs are shown in *parenthesis*. Note the difference in scaling of the *y*-axis compared with Fig. 5

In the thyroid, 25 genes were regulated at all investigated times of day (Fig. 4). Around half of these genes belonged to the Mus musculus species-specific kallikrein subfamily *Klk1b*. Basically, all genes showed a similar differential expression pattern with down-regulation at 9:00 and up-regulation at 12:00 and 15:00 (Fig. 4). The *Mat2a* gene (encoding the methionine adenosyltransferase II alpha) was the only exception showing an inverse pattern with up-regulation at 9:00 and down-regulation at later time points. Interestingly, *Mat2a* was also among the few genes showing comparatively low fold-change values. In general, the strength of down-regulation was more pronounced than up-regulation; 24 transcript probes showed a fold-change of at least 20-fold, 13 transcript probes of at least 60-fold, and 5 transcript probes of around 100-fold or higher. In comparison, up-regulation was comparatively weak in all instances, i.e., below 20-fold.

**Fig. 5:**
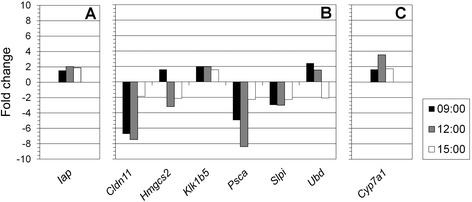
Transcripts regulated over time of day in kidney cortex and medulla, and liver. The fold-change of transcripts significantly regulated at all investigated times of day (9:00, *black*; 12:00, *gray*; 15:00, *white*) is shown for kidney cortex (**a**), kidney medulla (**b**), and liver (**c**). Up- and down-regulation is indicated by positive and negative values, respectively. Note the difference in scaling of the *y*-axis compared with Fig. 4

In contrast, few genes were regulated at all investigated times of day in the non-thyroid tissues, i.e., only one in the kidney cortex (Fig. 5a), six in the kidney medulla (Fig. 5b), and one in the liver (Fig. 5c). Moreover, none of these genes depicted the regulation pattern observed in the thyroid. In addition to the much lower number of genes, down-regulation at 9:00 occurred only for *Cldn11*, *Psca*, and *Slpi* in the kidney medulla. Fold-change values in the non-thyroid tissues were decidedly lower compared with the thyroid; up-regulation ranged from 1.5 to 3.5 fold-change, and down-regulation ranged from −1.9 to −8.4 fold-change. In both kidney cortex (Fig. 5a) and liver (Fig. 5c), the single detected gene (*Iap* and *Cyp7A1*, respectively) was up-regulated at all times of day with a maximum fold-change at 12:00. In the kidney medulla, *Cldn11*, *Psca*, and *Slpi* were consistently down-regulated, and *Klk1b5* was consistently up-regulated, while *Hmgcs2* and *Ubd* were the only genes that showed changes in the direction of regulation (Fig. 5b). In the lungs and spleen, no transcripts were differentially regulated at the investigated times of day (*cf*. Fig. 2).

Significant regulation of circadian clock genes was very low with only few regulation instances of *Per1*, *Ppara*, and *Ccnd1* in the liver or thyroid (Additional file 1: Table S1).

### Signature gene regulation

**Fig. 6:**
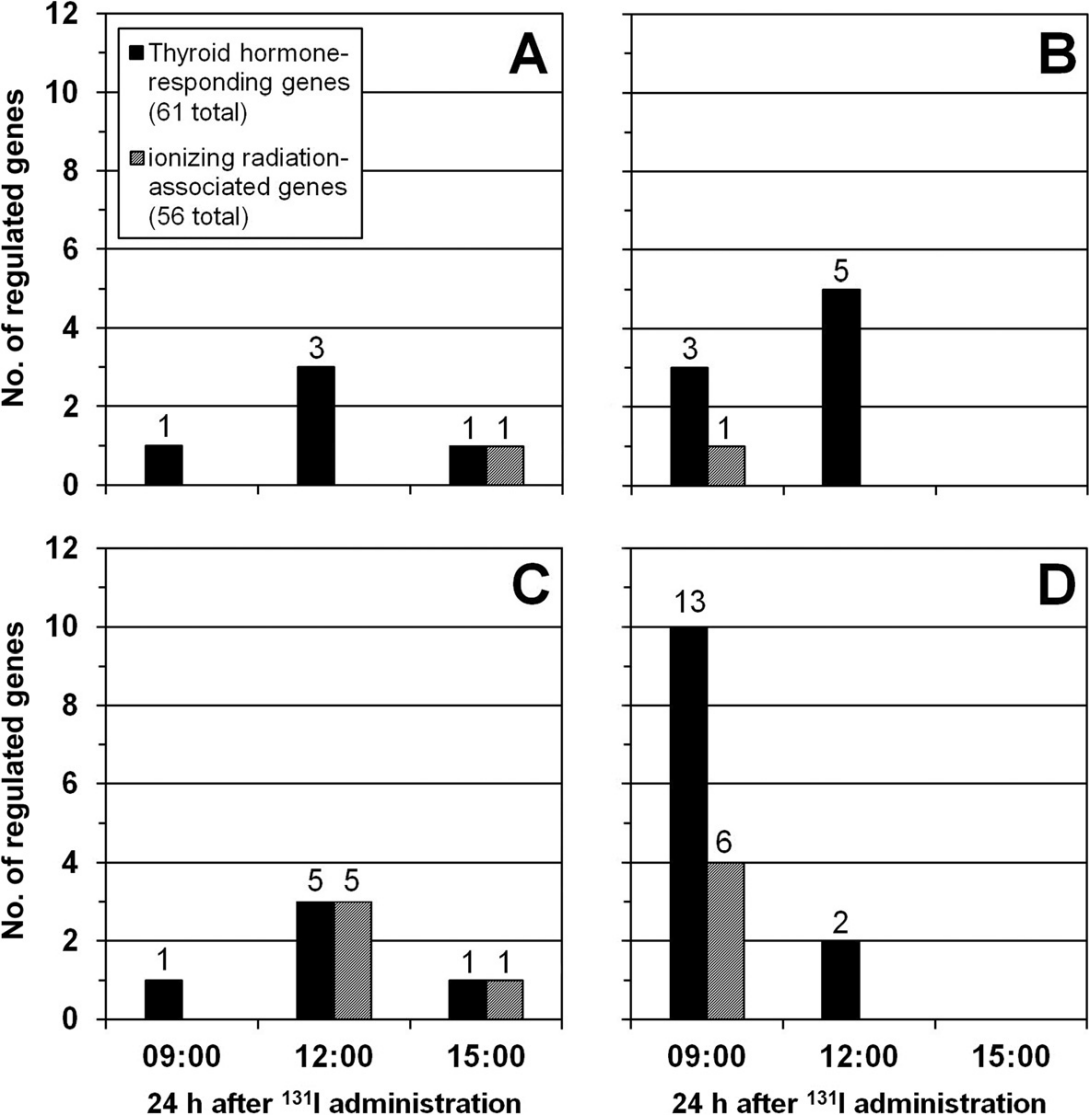
Signature gene regulation in kidney cortex and medulla, liver, and thyroid. The number (*no.*) of significantly regulated signature genes (thyroid hormone (TH)-responding, *black*; ionizing radiation (IR)-associated, *gray*) is shown for the kidney cortex (**a**), kidney medulla (**b**), liver (**c**), and thyroid (**d**) at respective time of day after 24 h following i.v. administered 90 kBq ^131^I. The number of significantly detected probes within a gene signature are stated above *bars*. No associated responses were observed in the lungs and spleen (not shown)

The impact of ionizing radiation and thyroid-dependent effects on transcriptional regulation was evaluated using IR-associated and TH-responding gene signatures. Signature genes were regulated in the kidney cortex (Fig. 6a) and medulla (Fig. 6b), liver (Fig. 6c), and thyroid (Fig. 6d). In these tissues, the number of TH-responding genes usually exceeded or at least equaled that of IR-associated genes at all investigated times of day. In the lungs and spleen, however, significant regulation was not observed for either signature (data not shown).

The highest number of regulated signature genes was seen in the thyroid at 9:00 with 10 TH-responding genes, which represented a pronounced global maximum. In the kidney cortex, kidney medulla, and liver, the highest number of regulated TH-responding signature genes was seen at 12:00, i.e., 3 h after the peak response in the thyroid. *Ccnd1* is reported in the literature as both IR-associated and TH-responding and was accordingly scored for both signatures.

Detection of multiple transcript probes for a signature gene was low and only observed in the liver (12:00) and thyroid (9:00); splicing variants were not detected (Additional file 2: Table S2 and Additional file 3: Table S3). Considering all investigated tissues, IR-associated transcripts had a mean (absolute) fold-change of 2.4 with a minimum of 1.5 and maximum of 7.2; most transcripts were down-regulated (Additional file 2: Table S2). TH-responding transcripts had a mean (absolute) fold-change of 4.0 with a minimum of 1.5 and a maximum of 41; around half of the transcripts were down-regulated (Additional file 3: Table S3).

### Diurnal variation in transcript-associated cellular function

**Fig. 7:**
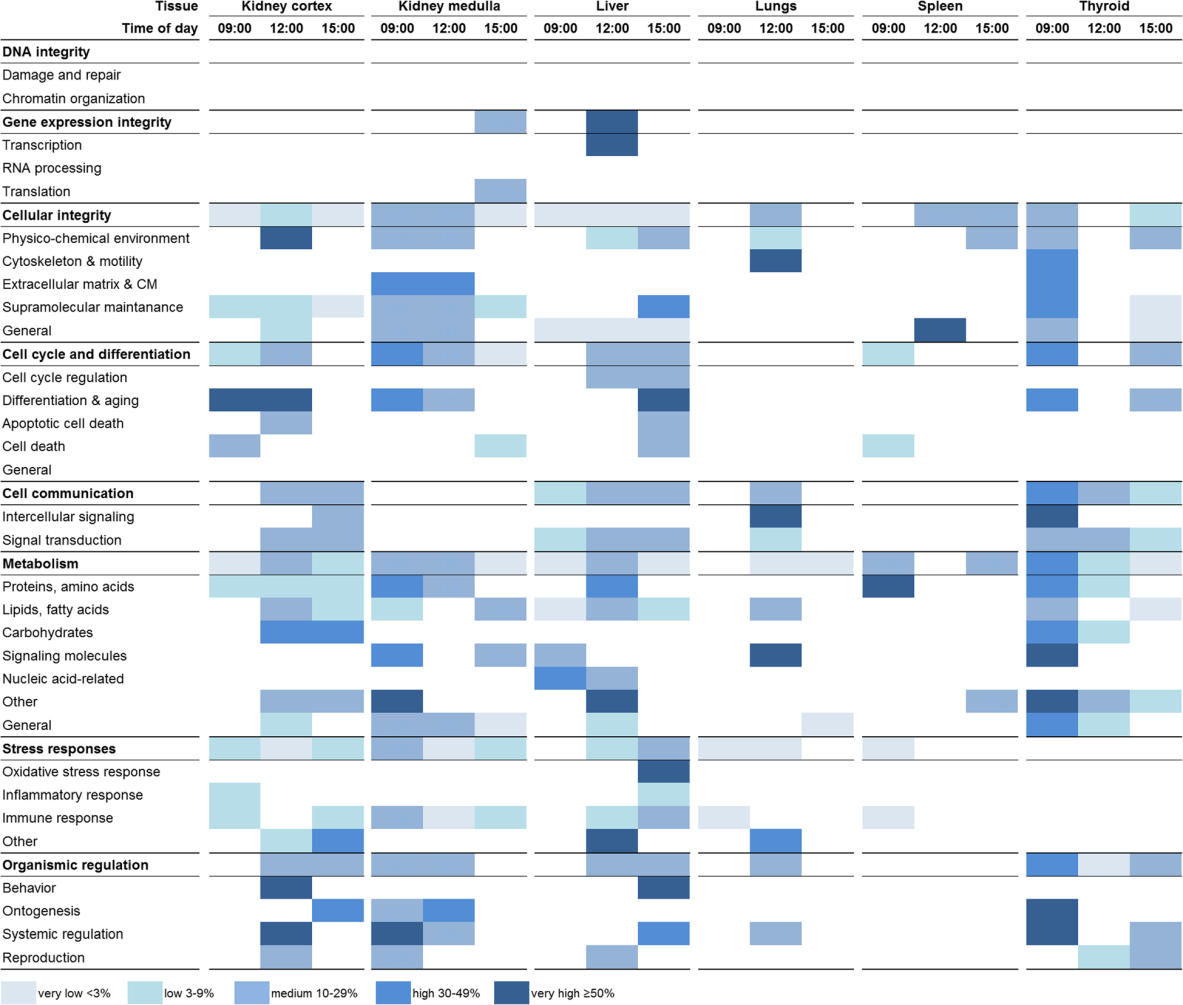
Heat map of enriched biological processes categorized after cellular function. Biological processes were enriched from significantly regulated transcripts (based on Gene Ontology (GO) terms) and then grouped into categories and subcategories of associated cellular function based on GO ancestor charts (http://geneontology.org). The percentage of scored vs. filtered transcripts is shown as very low <3 %, low 3–9 %, medium 10–29 %, high 30–49 %, and very high ≥50 %, and colored as *very light blue*, *light blue*, *blue*, *dark blue*, and *very dark blue*, respectively

Diurnal variation influenced the regulatory profile of transcript-associated cellular functions through the day (Fig. 7). The extent of regulation and the type of regulated subcategory generally differed between investigated times of day for each tissue. Comparison of regulation profiles between tissues at a certain time of day indicated tissue-specificity of responses to ^131^I administration.

In the thyroid, the regulatory profile at 9:00 appeared to be more similar to 15:00 than to 12:00 in terms of regulated subcategories, in particular regarding *cellular integrity* and *cell cycle and differentiation*. However, the overall regulation intensity (% regulation of scored vs. filtered transcripts) appeared to be more similar between 12:00 and 15:00, which was in agreement with the similar level of total transcript regulation at both times of day (*cf.* Fig. 1).

The kidney cortex, kidney medulla, and liver displayed a large spread among affected cellular functions through the day. In each of these tissues, few subcategories were regulated at all investigated times of day and regulation usually differed in intensity, thus supporting the trend of diversified cellular responses in dependence of time of day. Although only few transcripts were regulated in the lungs and spleen (Fig. 2), the associated cellular function differed distinctly through the day. In the lungs, for instance, biological processes for maintenance of *cellular integrity* or *cell communication* were only regulated at 12:00; moreover, no similarity was seen between the investigated times of day when regarding subcategories for *metabolism* or *stress responses*. Similarly in the spleen, the regulatory profile differed distinctly through the day on the subcategory level.

In all investigated tissues, no impact on *DNA integrity* was observed, which represented a striking common feature. Similarly, *gene expression integrity* was not regulated in most tissues except for the kidney medulla and liver, which showed only one subcategory response at 15:00 and 12:00, respectively. Furthermore, the kidney medulla and spleen also did not show any impact on biological processes related to *cell communication*. Lack of response was also observed for *cell cycle and differentiation* in the lungs, *stress responses* in the thyroid, and *organismic regulation* in the spleen.

## Discussion

^131^I exhibits differential uptake in the body where the majority of injected activity is taken up by and retained in the thyroid gland [18, 20]. The difference in absorbed dose over 24 h between the thyroid and the investigated non-thyroid tissues was substantial, i.e., 5.9 Gy vs. 0.75–2.2 mGy. The injected ^131^I activity and thus tissue-specific absorbed dose, however, were kept constant at all investigated times of day. Variation in circadian cycling of the transcriptome and proteome of mouse tissues in vivo has been demonstrated before, for instance in the liver [7, 26]. However, to the best of our knowledge, diurnal variation of genome-wide transcriptional responses has not been described in context with intravenously administered radionuclides in vivo. Regarding ^131^I administration, there are two aspects to be considered: first, differences in tissue-specific absorbed dose due to differential uptake, and second, systemic relation between a (dominantly exposed) regulatory tissue and (less exposed) target tissues. As we discussed in a previous study, transcriptional regulation in the liver, lungs, spleen, and cortical and medullary kidney tissues is not understood as exclusively induced by IR exposure from ^131^I in each tissue, but—to varying extent depending on tissue type—influenced by systemic factors originating from the thyroid gland [11]. The temporal dimension of observed variation in total transcriptional regulation further supported this reasoning; a peak response was observed at 9:00 in the thyroid and 3 h later in the kidney cortex, kidney medulla, and spleen. In contrast, the lungs and spleen did not show a pronounced maximum in total transcriptional regulation, and moreover, only few transcripts were regulated in both tissues. The lungs are the essential respiratory organ, and their main function, i.e., the intake of oxygen and the release of carbon dioxide, plays a large role for cellular metabolism. The spleen is not a vital organ but performs important functions for filtering blood and recycling iron, and it is further involved in the immune system as part of the mononuclear phagocyte system. However, tissue metabolism and turnover in both organs is low compared with the kidneys and liver. Overall, the differences in (total) transcriptional regulation between non-thyroid tissues are thought to reflect the relative metabolic activity and turnover in the tissues. Furthermore, these findings indicated that the lungs and spleen may be less likely affected by IR-induced damage in the thyroid than the kidneys or liver, which is an important finding for risk assessment.

In a previous study using a differential exposure setting with ^211^At, we hypothesized that thyroid-dependent systemic factors have an impact on target tissue responses [11]. In a follow-up study, we analyzed the relative impact of TH- or IR-induced gene regulation using a comprehensive panel of literature-based signature genes for each inducer [Langen B, Rudqvist N, Helou K, Forssell-Aronsson E: Microarray studies on ^211^At administration in BALB/c nude mice indicate systemic effects on transcriptional regulation in non-thyroid tissues, submitted]. In the present study, we investigated robustness of signature gene analysis with regard to diurnal variation. For all investigated times of day, responses in TH-responding signature genes were generally stronger than in IR-associated signature genes, although the extent (i.e., number of significantly regulated genes and respective fold-change values) varied between the investigated times of day. Nevertheless, the consistent trend in signature gene regulation, meaning a general dominance of TH-associated signature genes, was reproduced through the day, which validated this analytical endpoint with regard to circadian rhythm.

Diurnal variation was observed for all features of transcriptional regulation, i.e., number of regulated transcripts, fold-change value, and direction of regulation. Moreover, associated cellular functions of differentially regulated transcripts varied through the day in all investigated tissues. Interestingly, differential transcript regulation that was shared between two times of day after ^131^I administration was generally lower than differential regulation in the absence of IR exposure, i.e., only as an effect of diurnal change of regulation. Taken together, the presented data suggest that circadian rhythm affected the manner in which tissues responded to IR-induced damage. Considering the hypothesis of thyroid-dependent effects in the investigated non-thyroid tissues [11], the extent of the overall transcriptional responses appeared to correlate with basic metabolic turnover in non-thyroid tissues. Unfortunately, it could not be deduced from this data to what extent transcriptional regulation was induced by IR exposure in each tissue or by systemic factors originating from the thyroid. Nevertheless, the general dominance of TH-responding genes over IR-associated genes suggested that a significant contribution from systemic factors could be considered likely.

A distinct pattern was observed for genes that were regulated in the thyroid at 9:00, 12:00, and 15:00; the vast majority was strongly down-regulated at 9:00 and comparatively mildly up-regulated at later time points. This finding underlined that certain genes are regulated upon IR exposure irrespective of time of day, but that the quality of induced regulation is influenced by circadian oscillation of gene expression, which may have consequences for cellular outcome. A large number of these genes belonged to the kallikrein subfamily *Klk1b*, which has been proposed as molecular biomarkers for ^131^I and ^211^At exposure in the thyroid [27]. The present study demonstrated that transcriptional regulation of *Klk1b* genes in responses to 5.9 Gy absorbed dose from ^131^I varied distinctly through the day; nevertheless, significant regulation could be detected at any of the investigated times. Bearing in mind the time-dependent pattern, this study validated significant detection of *Klk1b* transcripts as biomarkers from morning to afternoon. Nevertheless, regulation patterns of potential biomarkers should be characterized throughout the day in order to assess whether changes in expression are induced by irradiation condition or circadian rhythmicity. Accordingly, it is advised to match the time of day in, e.g., long-term studies and to repeat experiments at different times of day to avoid bias from diurnal variation.

The phasing of circadian rhythms can be shifted by various stressors [28–30]. Ionizing radiation, for instance, can phase advance the circadian clock as has been demonstrated for instance in rat fibroblasts using γ-radiation in the range of 0.5–12 Gy [30]. It is thus possible that IR-induced phase shifting occurred in the thyroid due to the high absorbed dose of 5.9 Gy. This potential effect would have implications for, e.g., the time of day when maximum transcript regulation was observed. Significant up- or down-regulation in circadian clock genes was generally not detected, which might indicate that phase shifting did not occur. However, phase shifting is difficult to demonstrate in this set-up, i.e. with analysis at a single 24 h time-point.

It has recently been demonstrated that DNA damage response pathways and cell cycle progression are under circadian control [31–33]. Moreover, a significant difference in mRNA levels of DNA damage inducible genes in mouse blood and bone marrow was demonstrated between diurnal and nocturnal irradiation with 0.5 Gy X-ray in vivo [34]. In the present study, enriched biological processes did not show responses for DNA damage and repair for chromatin organization. A (very) low absorbed dose level is not expected to result in up-regulation of the DNA damage recognition and repair machinery on the transcriptional level. While this was the case for the non-thyroid tissues, an absorbed dose of 5.9 Gy in thyroid tissue may result in transcriptional regulation of DNA damage and repair-related genes. However, no responses were detected in maintenance of *DNA integrity* in thyroid tissue. This may be explained, in part, by continuous low-dose-rate irradiation which may not suffice to induce an acute damage and repair response; in general, the absorbed dose of 5.9 Gy over 24 h constituted a (very) low-dose rate of a few mGy per minute, although it should be noted that the dose rate of ^131^I in thyroid varies over time [20]. Another factor influencing detection of DNA damage and repair-related processes may be convolution of hit vs. non-hit cells (and multi-hit cells) in microarray data. Individual cells that were subjected to a sufficiently high damage burden may induce transcriptional regulation of respective genes, but the expression changes may be masked in the mixed (homogenized) cell population. Nevertheless, analysis of DNA damage and repair responses to ionizing radiation exposure, specifically in longitudinal studies, should consider differences in response with regard to time-of-irradiation and, if possible, account for circadian oscillation in the experimental design.

Overall, these findings suggest that the timing of clinical studies or therapeutic schedules can have a pronounced effect on obtained results or therapeutic outcome, since circadian rhythm is an intrinsic factor in cellular and physiological regulation in humans as well. However, since mice are nocturnal animals but are usually treated during the day in experiments, it may be advisable to phase shift animals in pre-clinical studies to better match human physiology in the clinical setting.

## Conclusions

In this study, we demonstrated diurnal variation at 9:00, 12:00, and 15:00 of transcriptional responses to intravenously administered ^131^I in the kidney cortex and medulla, liver, and thyroid. In contrast, differential regulation in the lungs and spleen was generally low irrespective of time of day at the investigated exposure condition. Diurnal variation was demonstrated for several features of transcriptional regulation, i.e., total number of significantly regulated transcripts, fold-change and direction of regulation, and transcript-associated cellular function. Despite diurnal variation, main results on ^131^I-induced transcriptional regulation were reproduced irrespective of time of day, which validated the analytical endpoints used for radiogenomic biomarker discovery in vivo.

Overall, we conclude that time of day can play both a minor and major role for gene expression analysis, which appears to be dependent on the investigated gene set and on the quality and strength of induced responses. The relative impact that circadian rhythm has on the biological endpoint studied may be dependent on absorbed dose and differ between low-dose and high-dose regimens. Depending on the exposure condition, daytime-of-treatment or daytime-of-measurement may have a larger effect on gene expression than the actual ionizing radiation exposure—or the effect of circadian rhythm may be negligible. Thus, we advocate that circadian rhythm should be considered as an experimental variable in radiation research and that “circadian robustness” should be validated for the biological or analytical endpoint under investigation.

## Additional files

Additional file 1: Table S1. Circadian rhythm core genes and significant regulation. Core circadian clock genes as reviewed by Kelleher FC, Rao A, Maguire A. Circadian molecular clocks and cancer. Cancer Lett. 2014;342:9–18. Review. (DOCX 20 kb) Additional file 2: Table S2. Significantly regulated ionizing radiation-associated genes. (DOCX 20 kb) Additional file 3: Table S3. Significantly regulated thyroid hormone-responding genes. (DOCX 23 kb)
