# Supplementary material for: Circadian rhythm influences genome-wide transcriptional responses to 131I in a tissue-specific manner in mice
Source: EJNMMI Res. 2015 Dec 15;5:75. doi: 10.1186/s13550-015-0150-y (PMC4679710; doi:10.1186/s13550-015-0150-y)
Supplement: Additional file 1: Table S1. — Circadian rhythm core genes and significant regulation. Core circadian clock genes as reviewed by Kelleher FC, Rao A, Maguire A. Circadian molecular clocks and cancer. Cancer Lett. 2014;342:9–18. Review. (DOCX 20 kb) [file 13550_2015_150_MOESM1_ESM.docx]

SUPPLEMENTAL TABLE 1. Circadian rhythm core genes and significant regulation

Gene symbol		Gene name	Significantly regulated in sample cohort			
Mouse	Human homolog		Tissue	Time-of-day	Regulation	Fold-change
Cry1	*CRY1*	cryptochrome 1 (photolyase-like)	*none*			
Cry2	*CRY2*	cryptochrome 2 (photolyase-like)	*none*			
Per1	*PER1*	period circadian clock 1	Liver	15:00	down	-1.7
Per2	*PER2*	period circadian clock 2	*none*			
Per3	*PER3*	period circadian clock 3	*none*			
Clock	*CLOCK*	circadian locomotor output cycles kaput	*none*			
Arntl	*ARNTL (BMAL1)*	aryl hydrocarbon receptor nuclear translocator-like	*none*			
Npas2	*NPAS2*	neuronal PAS domain protein 2	*none*			
Rora	*RORα*	RAR-related orphan receptor alpha	*none*			
Nr1d1	*NR1D1 (hRev)*	nuclear receptor subfamily 1, group D, member 1	*none*			
Ppara	*PPARα*	peroxisome proliferator activated receptor alpha	Liver	15:00	down	-1.5
			Thyroid	9:00	up	2.0
Pparag	*PPARγ*	peroxisome proliferator activated receptor gamma	*none*			
Myc	*MYC (c-MYC)*	myelocytomatosis oncogene	*none*			
Wee1	*WEE1*	WEE 1 homolog 1 (S. pombe)	*none*			
Ccnd1	*cyclin D*	cyclin D1	Liver	12:00	down	-1.7
			Thyroid	9:00	down	-1.9
Cdkn1a	*CDKN1A (P21)*	cyclin-dependent kinase inhibitor 1A (P21)	*none*			

Core circadian clock genes as reviewed by

Kelleher FC, Rao A, Maguire A. Circadian molecular clocks and cancer. Cancer Lett. 2014;342:9–18. Review.
