# Supplementary material for: Circadian rhythm influences genome-wide transcriptional responses to 131I in a tissue-specific manner in mice
Source: EJNMMI Res. 2015 Dec 15;5:75. doi: 10.1186/s13550-015-0150-y (PMC4679710; doi:10.1186/s13550-015-0150-y)
Supplement: Additional file 2: Table S2. — Significantly regulated ionizing radiation-associated genes. (DOCX 20 kb) [file 13550_2015_150_MOESM2_ESM.docx]

**SUPPLEMENTAL TABLE 2. Significantly regulated ionizing radiation-associated genes**

| Mouse tissue | Time point | Ionizing radiation associated genes | | | |
|---|---|---|---|---|---|
| | | Gene symbol | Probe ID | Transcript ID | Fold-change |
| Kidney cortex | 09:00 | *none* | | | |
| | 12:00 | *none* | | | |
| | 15:00 | *Gja1* | ILMN_1244291 | ILMN_217762 | -2.0 |
| Kidney medulla | 09:00 | *Gja1* | ILMN_1244291 | ILMN_217762 | -1.5 |
| | 12:00 | *none* | | | |
| | 15:00 | *none* | | | |
| Liver | 09:00 | *none* | | | |
| | 12:00 | *Ccnd1* | ILMN_1221503 | ILMN_210028 | -1.8 |
| | | | ILMN_2601471 | ILMN_210028 | -1.6 |
| | | | ILMN_2669793 | ILMN_210028 | -1.6 |
| | | *Ephx2* | ILMN_1226688 | ILMN_223103 | -1.9 |
| | | *Gadd45g* | ILMN_2903945 | ILMN_222120 | 2.1 |
| | 15:00 | *Tgfbr2* | ILMN_2760979 | ILMN_221064 | 1.5 |
| Lungs | 09:00 | *none* | | | |
| | 12:00 | *none* | | | |
| | 15:00 | *none* | | | |
| Spleen | 09:00 | *none* | | | |
| | 12:00 | *none* | | | |
| | 15:00 | *none* | | | |
| Thyroid | 09:00 | *Ccnd1* | ILMN_1221503 | ILMN_210028 | -1.9 |
| | | | ILMN_2601471 | ILMN_210028 | -1.9 |
| | | *Ccng1* | ILMN_2500276 | ILMN_232357 | 1.7 |
| | | *Gjb2* | ILMN_1227148 | ILMN_210347 | -4.6 |
| | | | ILMN_2999627 | ILMN_210347 | -7.2 |
| | | *Plcg2* | ILMN_2601833 | ILMN_210061 | -2.1 |
| | 12:00 | *none* | | | |
| | 15:00 | *none* | | | |
