# Supplementary material for: Circadian rhythm influences genome-wide transcriptional responses to 131I in a tissue-specific manner in mice
Source: EJNMMI Res. 2015 Dec 15;5:75. doi: 10.1186/s13550-015-0150-y (PMC4679710; doi:10.1186/s13550-015-0150-y)
Supplement: Additional file 3: Table S3. — Significantly regulated thyroid hormone-responding genes. (DOCX 23 kb) [file 13550_2015_150_MOESM3_ESM.docx]

SUPPLEMENTAL TABLE 3. Significantly regulated thyroid hormone-responding genes

Mouse tissue	Time point	Thyroid hormone responding genes			
		Gene symbol	Probe ID	Transcript ID	Fold-change
Kidney cortex	09:00	*Gsta1*	ILMN_2979237	ILMN_196592	1.5
	12:00	*Gsta1*	ILMN_2979237	ILMN_196592	-2.3
		Pck1	ILMN_1213632	ILMN_238641	-2.6
		Thrsp	ILMN_1256775	ILMN_209127	2.3
	15:00	*Thrsp*	ILMN_1256775	ILMN_209127	-3.0
Kidney medulla	09:00	*Gsta1*	ILMN_2979237	ILMN_196592	1.6
		Mbp	ILMN_3011353	ILMN_250782	-1.6
		Pck1	ILMN_1213632	ILMN_238641	1.6
	12:00	*Gsta1*	ILMN_2979237	ILMN_196592	-2.2
		Lmo2	ILMN_2767605	ILMN_223680	-1.7
		Mbp	ILMN_3011353	ILMN_250782	-1.6
		Pck1	ILMN_1213632	ILMN_238641	2.6
		Slc16a6	ILMN_1258950	ILMN_233199	1.6
	15:00	*none*			
Liver	09:00	*Slc16a6*	ILMN_1258950	ILMN_233199	-1.8
	12:00	*Ccnd1*	ILMN_1221503	ILMN_210028	-1.8
			ILMN_2601471	ILMN_210028	-1.6
			ILMN_2669793	ILMN_210028	-1.6
		Cyp7a1	ILMN_2604383	ILMN_210317	3.5
		Gsta1	ILMN_2979237	ILMN_196592	-2.2
	15:00	*Cyp7a1*	ILMN_2604383	ILMN_210317	1.7
Lungs	09:00	*none*			
	12:00	*none*			
	15:00	*none*			
Spleen	09:00	*none*			
	12:00	*none*			
	15:00	*none*			
Thyroid	09:00	*Atp2a1*	ILMN_2666864	ILMN_216061	41
		Ccnd1	ILMN_1221503	ILMN_210028	-1.9
			ILMN_2601471	ILMN_210028	-1.9
		Cd44	ILMN_3114585	ILMN_245439	-1.6
		Egf	ILMN_2684104	ILMN_217509	-31
		Lmo2	ILMN_2767605	ILMN_223680	-2.3
		Pck1	ILMN_1213632	ILMN_238641	2.3
		Prkag2	ILMN_3161626	ILMN_221101	1.7
		Slc16a6	ILMN_1258950	ILMN_233199	-1.9
		Slc2a1	ILMN_1258159	ILMN_215238	-1.9
		Vldlr	ILMN_1218264	ILMN_188056	2.2
			ILMN_2515601	ILMN_188056	2.2
			ILMN_2796472	ILMN_188056	2.2
	12:00	*Egf*	ILMN_2684104	ILMN_217509	4.2
		Pck1	ILMN_1213632	ILMN_238641	2.0
	15:00	*none*			
